# Metabolite AutoPlotter - an application to process and visualise metabolite data in the web browser

**DOI:** 10.1186/s40170-020-00220-x

**Published:** 2020-07-10

**Authors:** Matthias Pietzke, Alexei Vazquez

**Affiliations:** 1 Cancer Research UK Beatson Institute, Switchback Road, Bearsden, Glasgow, G61 1BD UK; 2 Institute of Cancer Sciences, University of Glasgow, Glasgow, UK

**Keywords:** Metabolites, Metabolomics, Processing, Visualisation, Graphs, Plots, Automatic

## Abstract

**Background:**

Metabolomics is gaining popularity as a standard tool for the investigation of biological systems. Yet, parsing metabolomics data in the absence of in-house computational scientists can be overwhelming and time-consuming. As a consequence of manual data processing, the results are often not analysed in full depth, so potential novel findings might get lost.

**Methods:**

To tackle this problem, we developed Metabolite AutoPlotter, a tool to process and visualise quantified metabolite data. Other than with bulk data visualisations, such as heat maps, the aim of the tool is to generate single plots for each metabolite. For this purpose, it reads as input pre-processed metabolite-intensity tables and accepts different experimental designs, with respect to the number of metabolites, conditions and replicates. The code was written in the R-scripting language and wrapped into a shiny application that can be run online in a web browser on https://mpietzke.shinyapps.io/autoplotter.

**Results:**

We demonstrate the main features and the ease of use with two different metabolite datasets, for quantitative experiments and for stable isotope tracing experiments. We show how the plots generated by the tool can be interactively modified with respect to plot type, colours, text labels and the shown statistics. We also demonstrate the application towards ^13^C-tracing experiments and the seamless integration of natural abundance correction, which facilitates the better interpretation of stable isotope tracing experiments. The output of the tool is a zip-file containing one single plot for each metabolite as well as restructured tables that can be used for further analysis.

**Conclusion:**

With the help of Metabolite AutoPlotter, it is now possible to simplify data processing and visualisation for a wide audience. High-quality plots from complex data can be generated in a short time by pressing a few buttons. This offers dramatic improvements over manual analysis. It is significantly faster and allows researchers to spend more time interpreting the results or to perform follow-up experiments. Further, this eliminates potential copy-and-paste errors or tedious repetitions when things need to be changed. We are sure that this tool will help to improve and speed up scientific discoveries.

## Background

Data analysis for metabolomics can typically be separated into two parts. In the first part, often termed as pre-processing, the raw measurements are converted into readouts. This includes reading the mass-spectrometer raw files, peak detection and integration, metabolite identification and alignment of metabolites over several measurements [1, 2]. In the second part of the analysis, researchers need to convert readouts into biological insights. While there are tools to carry on the identification and quantification of metabolites [3], we are still missing user-friendly interfaces to perform statistics and visualise the data. These tasks typically include the association of measurements to conditions, grouping and averaging of replicate measurements, correction of the intensities by cell counts or internal standards and finally visualising the results.

It is difficult to define a universal solution due to different data structures generated with different analytic tools, different experimental setups and finally different personal visual preferences. As a consequence, we found others and ourselves repeating similar steps manually over and over again, extracting data for selected metabolites to be imported in graphic tools such as GraphPad Prism or plotting metabolite intensities with Microsoft Excel. However, manual data processing is extremely time-consuming and prone to errors.

To automate these steps, we developed Metabolite AutoPlotter, a web-based application for the analysis of metabolomics data, conveniently automatizing the steps in metabolomics data processing, leading to well-structured tables and graph outputs for every metabolite in the dataset. The graphs can be customised with regard to plot type, colours, text labels and size among other features. Additionally, statistical tests can be conducted and the results added to the graphs. The most important features are described in detail in the following sections.

## Methods

### Implementation

Metabolite AutoPlotter is written in R [4] and wrapped into a web-application with the “shiny” package [5]. Data manipulation is realised mainly with tools from the “tidyverse” package [6], the plotting is realised with “ggplot2” [7] and for the individual data points, “ggbeeswarm” is used. For reading and writing Excel files, “readxl” and “writexl” are used; for creating the report file, we use “officer”. Statistics is implemented with “ggpubr”, containing a wrapper for the “ggsignif” package. Summary pages are created using “gridExtra”, the palettes are used from the “pals” package and colour blind simulation is performed using “colorspace”. The appearance of the application is improved with “shinythemes”, “shinycssloaders”, “shinyWidgets” and “shinyalert”. For zipping of the results, the “zip” package is used. All packages and the R-version are routinely updated.

### Generation of metabolite data for a quantitative experiment

For the quantitative demo data, we extracted intracellular metabolites from 5 different tissues (brain, liver, kidney, spleen and pancreas) from a ^13^C-tracing experiment with ^13^C-methanol in four different mice [8]. Metabolite extraction and LC-MS measurement were performed as described in [9]. Only the unlabelled metabolites are used here, to show the differences between the tissues. Peak areas were extracted using TraceFinder-software (Thermo Fisher), by comparing the mass to charge ratio (m/z) and the retention time against a custom library prepared with authentic standards. The dataset contains 99 metabolites measured for 3 time-points in 4 biological replicates.

### Generation of metabolite data for tracing experiment

This dataset was generated exclusively for the purpose of showing the tracing features of this tool. For this, HCT116 cells were incubated for four different time points (1 h, 3 h, 6 h and 24 h) in Dulbecco’s modified Eagle’s medium (DMEM) either containing u-^13^C_6_-Glucose (17mM) or u-^13^C_5_-glutamine (2 mM) in triplicate wells. Intracellular metabolites were extracted and measured by LC-MS again as described in [9], and 38 metabolites were extracted using TraceFinder-software (Thermo Fisher).

### Implementation of default colours

As default colours for the plots, we use the schemes developed by Paul Tol (https://personal.sron.nl/~pault/) and their implementation in the pals-package. This delivers a harmonic appearance while simultaneously maintaining a fair amount of separation for people with colour-impaired vision. For quantitative experiments, the user can define its own colours in the sample table either in hex-notation (e.g. #FFAA25) or using the predefined colour names for R. For tracing experiments, the colours are hardcoded to guarantee an identical appearance between experiments. Here, the user can choose between the short scale, a modification of the tol-rainbow, with saturation and value increased by 20%, and the long scale is based on the stepped rainbow.

## Results

### Data import

Metabolite AutoPlotter reads quantified data containing the identified metabolites and their intensities, generated elsewhere, e.g. with manufacturer’s software or other software tools for peak detection and metabolite identification. Processed data need to include at least the metabolite names, unique sample IDs (as filenames) and the intensity (area or height) and can be imported as Microsoft Excel format (xls/xlsx), comma-separated files (csv) or other text formats. We implemented direct imports for the 2 most common outputs generated in our institute for LC-MS data (TraceFinder & Compound Discoverer, both from Thermo Fisher Scientific). Alternatively, data can be imported in a more generalised way either as a list or as a matrix, to allow the import of data generated with other software tools. A description of the data structures and expected columns is shown in Table 1.

**Table 1:** Data structure for the different input layouts

Input style	Layout	File types	Essential columns	Additional columns
Tracefinder	Both, metabolites and samples along rows	.csv or .xls(x)	Compound^1^, Filename, Area	Actual RT^2^, Formula^2+3^, Adduct^2^, m/z (Apex)^2^, m/z (Delta (ppm))^2^ or m/z (Delta)^2^
Compound Discoverer	Metabolites in rows, samples in columns	.csv or .xls(x)	Compound, RT [min]^4+5^, Filename as: "Area: "+ "filename"+".raw (F"number")" or "Norm. Area: "+ "filename"+".raw (F"number")"	Molecular Weight^2+5^ ,Mass^5^, Formula^3^
Compounds in Columns	Samples in rows (sample names in first column) metabolite names in first row (column 2-n)	.csv or .xls(x)	Samples in first column, name of column is ignored	Not allowed as they will be interpreted as metabolites
Other - List	Both, metabolites and samples along rows	.csv, .xls(x), tab-, comma, semicolon separated files	4 columns in defined order, describing the Metabolite^1^, RT^6^, Filename and Area	Will be ignored
Other - Matrix	Metabolites in rows, samples in columns (inverse of compounds in columns)		First 2 columns containing Metabolite^7^ and RT^6+8^, following columns containing the samples	Not allowed as they will be interpreted as metabolites

All imports need to have (only) one header row containing the column names. When Excel files are used, the data are expected in the first sheet. Remarks: 1-cannot contain empty values or duplicated names. 2-used for metabolite summary. 3-used for subtracting naturally occurring isotopes. 4-used to combine compound names with retention time (RT) as compound name typically contains duplicates. 5-used to label unknowns. 6-column RT can be empty but needs to be present. 7-Compounds can contain duplicate names as long as RT is supported to merge names and RT

### Adding sample descriptions

**Fig. 1:**
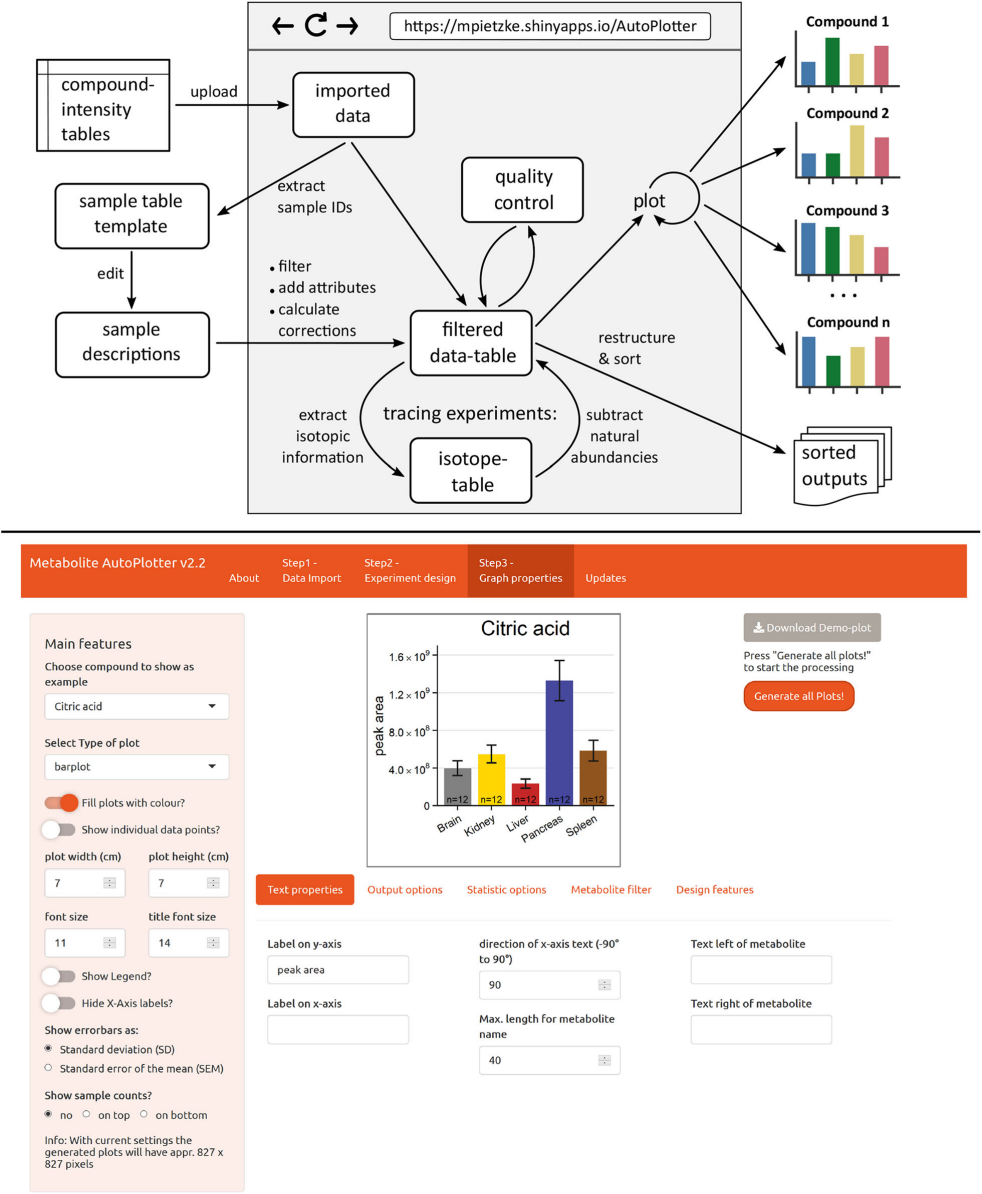
The visual design of the tool. Top: Scheme illustrating the workflow of the application. Bottom: Screenshot of the application, showing the plot-design step. The plot in the middle is interactively updated based on the settings defined by the user, allowing a high level of flexibility

Figure 1, top, illustrates the AutoPlotter workflow. Once the data was imported, it is converted to the internal data structure. A template for the sample table is generated and downloaded from within the app in order to define the conditions and the main properties used for the plotting. This includes the names of conditions shown on the plots, the order of the conditions and their colours. Samples can be normalised at this stage by cell counts or protein content. AutoPlotter offers two different strategies to do so. For the “absolute correction”, the metabolite intensities (peak areas) are divided by the supplied correction value. This can be used to express the results as “peak area per million cells” or “peak area per microgram of protein”, and is particularly useful when absolute quantities are used. Alternatively, a “relative correction” can be used. Here, the metabolite intensities are divided by the given correction value and then multiplied with the average of all correction values. As a result, the dimension of the metabolite intensities is maintained and the samples are equalised to similar levels. Consequently, this strategy is not recommended when differences between samples should be maintained, for example when different cell lines are used, differing in size and therefore in the harvested sample counts.

Further, different levels of replicates as measurement (~technical) replicates and experimental (~biological) replicates can be specified in the sample descriptions. In the latter case, the measurement replicates for each experimental replicate will be averaged and the averages will be used to present the data. Once the edited sample table is uploaded, it is saved together with the cleaned input data in the folder “Inputs” contained in the result folder. With these two files, the processing can be repeated at any time in the future.

### Data processing

The philosophy of the tool is to represent the uploaded data as original as possible. Missing values can occur in metabolomics experiments as a result of metabolites being absent in some samples or being present with a concentration below the detection limit. Consequently, missing values occur more frequently for low abundant compounds. Nevertheless, missing values can also appear independent of the concentration due to problems with the peak integration, as m/z not matching within the defined mass error due to coeluting compounds or retention time shifts outside the allowed window. Missing values are explicitly accepted by the tool and are further treated as “missing”, we do not impute missing values or fill missing values with zeros. Inputs as empty cells, “NA”, “NaN”, “N/F” and “N/A” are treated as missing. For the “Compounds in Columns” input, additionally, zeros can be treated as “missing” or kept as zero and hence as explicit missing. For “Compound Discoverer” or “Matrix” inputs, a threshold can be defined that need to be reached; below that threshold values are treated as missing. In line with this, we do not normalise, scale or transform the data. Although this is required for some further analysis (principal component analysis (PCA), independent component analysis (ICA), partial least square discriminatory analysis (PLS-DA) or clustering), this is not needed to show the data. The presence of missing values helps to identify poor measurements or noisy metabolites, while the addition of missing value imputation may gain false confidence.

At the step of data processing, metabolites can be normalised with internal standards. Normalisation can be performed before or after the external normalisation. The first case is suited for standards added during the extraction step, to compensate for variations during sample preparation and measurement. The latter case is useful when the intensities should be normalised to a detected metabolite. Similar to the external normalisation values can be normalised absolutely or relatively. Multiple metabolites can be selected and will be summed up, consequently, the normalisation is more affected by highly abundant metabolites. When there is not a single metabolite serving as a representative for the other compounds, samples can additionally be normalised with the sum of all peak areas (“Total Peak Sum”).

To further evaluate the quality of the data, we also include a quality control feature to evaluate the performance of the replicate measurements. For this, the relative fold changes for each metabolite between the replicates of the same condition are calculated and shown for all metabolites together. The means of all the metabolites should be centred around 1 when the replicates perform comparably. This strategy evaluates the quality of the samples based on all detected metabolites, is independent of outliers in single metabolites and missing values and works well for a small number of replicates. Samples that differ dramatically at this stage can be identified and removed conveniently before performing further analysis.

After the data are processed, structured tables are exported together with the plots. These tables can be used for further processing, statistical analysis or to import the data into other visualisation tools, e.g. GraphPad Prism. A detailed explanation of the exports is included in Supplement File 1, and is exported together with the results.

### Application to quantitative experiments

The most important and unique feature of the tool is to automatically generate one single graph for every compound in the dataset. As this highly depends on the user preferences, we included an interface that allows an interactive customisation of the plot design (Fig. 1, bottom).

**Fig. 2:**
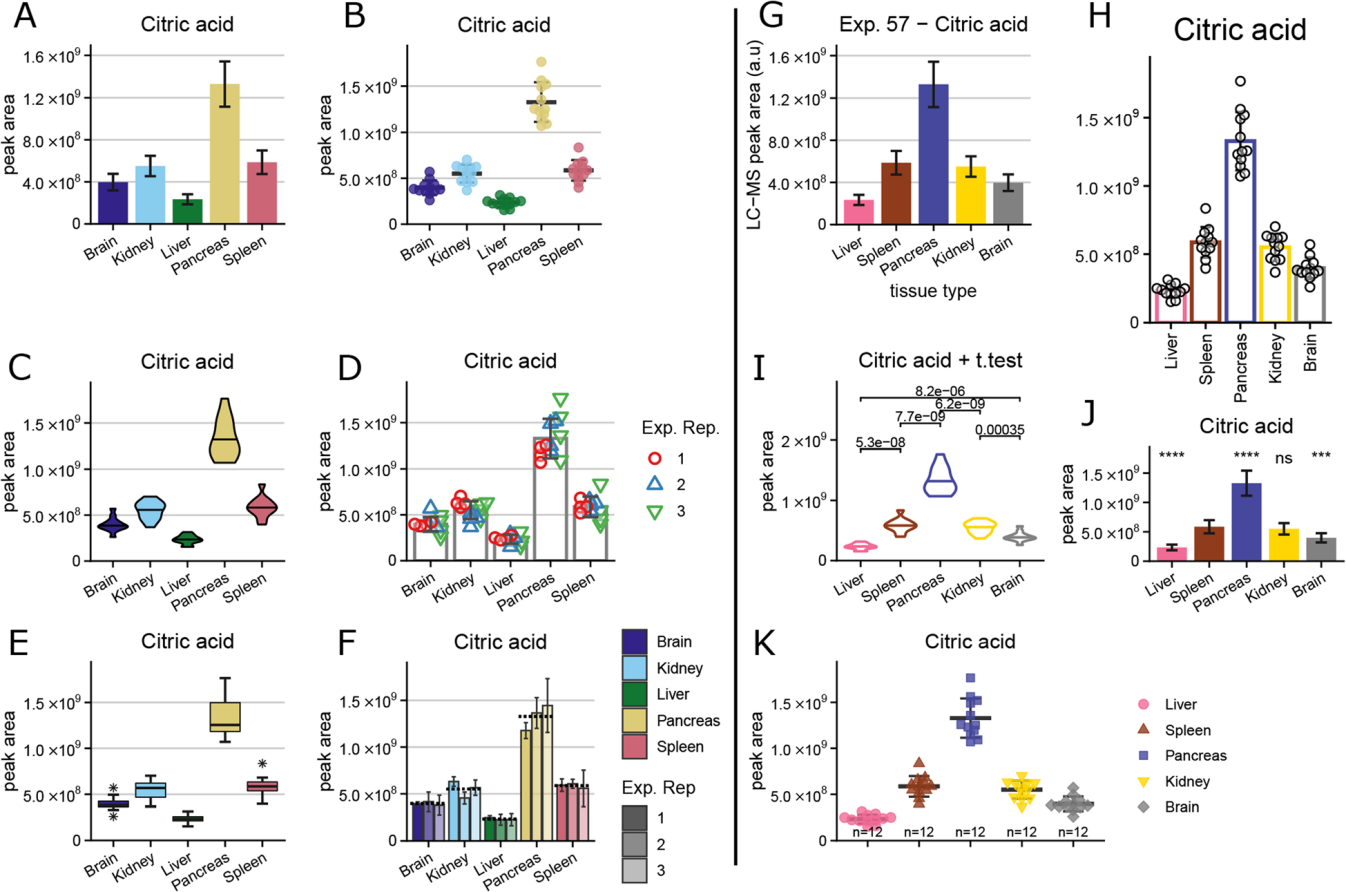
Illustration of plotting capabilities. **a**–**f** Standard plots included in the tool, with default parameters and colours. **a** Bar plot showing mean and standard deviation (SD). **b** Individual data-points with mean and SD. **c** Violin plot showing the distribution and the median (only available for at least 5 replicates). **d** Replication validation with points, showing the experimental replicates (only when experimental replicates are defined). **e** Boxplot (only available for at least 10 replicates). **f** Replication validation with bars, showing the experimental replicates (only when experimental replicates are defined). **g**–**j** Modifications on the plots allowing more flexibility, including user defined order and colours. **g** Modified text elements. **h** Hollow bars with added data points, removed grid lines, increased height and 90° direction on *x*-axis. **i** Hollow violin plot with statistics (*t* test with multiple pairwise comparisons). **j** Bar plot with decreased height and statistics (*t* test against spleen sample). **k** Points and error with increased width, legend instead of *x*-axis labels and added sample counts

Four different plot types are currently included in the application: bar plot, violin plot, box plot, and univariate scatterplots (Fig. 2a–c and e). The bar plots show the mean and the standard deviation, the same applies to the scatterplots that additionally show the individual measurements. The violin plots show the density distribution and the median, the box plots show the median, the 25th and 75th percentiles and the lowest/highest value within 1.5 times inter-quartile range from the box. Violin plots and box plots only make sense when enough data points are present, so they are only available when 5, respectively 10 replicate measurements per condition, are present in the dataset. When technical and experimental replicates are present, the user can additionally generate 2 different plots that allow for the comparison of the performance within the replicates (Fig. 2d, f).

### Plot personalisation

All plots can be further modified beyond the default settings (Fig. 2g–k). Already with the sample descriptions, the order of the conditions and their colours can be defined (Fig. 2, right vs. Fig. 2, left). Bar, box and violin plots can be generated with outlined colour instead of filled colour to reduce their weight and amount of ink used (Fig. 2h, i). Additionally, the individual data points can be overlaid over each of the plots (Fig. 2h). Furthermore, the dimensions of the plots (width and height), their resolution and the output format (jpg, pdf, png, svg) can be defined. Also, the text elements can be altered, this includes the sizes, the direction of the text along the *x*-axis, the axis titles for *x*- and *y*-axes. Additional text can be added on the left or the right side of the compound names to add some experimental descriptions. Simple statistics can be added as well; this can be either multiple pairwise comparisons or comparisons against one reference group, as *t* test (expecting equal variances) or Wilcoxon test. The results of the statistics can be shown as symbols or numeric values (Fig. 2i, j).

Once the design is defined, the user can click on “Generate all plots!” and a plot for every metabolite is saved as an image file with the metabolite name as the filename. Characters not allowed in filenames (e.g. :, ;, <, >) are converted automatically during this step. All results (the plots and the tables) are packed into a zip file that can be downloaded by the user.

Bar plots belong to the standard repertoire for data visualisation, as they are easily understood by anyone and can be effectively used to screen for differences due to the weight of the different colours. However, we want to point out that they are probably not the best representation in all cases. First of all, the data usually do not show continuous counts from zero to the end value, but multiple numbers with a similar distance away from zero. Secondly, they highly compress the data, hiding some underlying features of the data sets [10, 11]. Metabolite AutoPlotter includes different features to overcome this limitation, e.g., by overlaying the individual data points or using violin plots to better represent the distributions [12].

### Application to tracing experiments

**Fig. 3:**
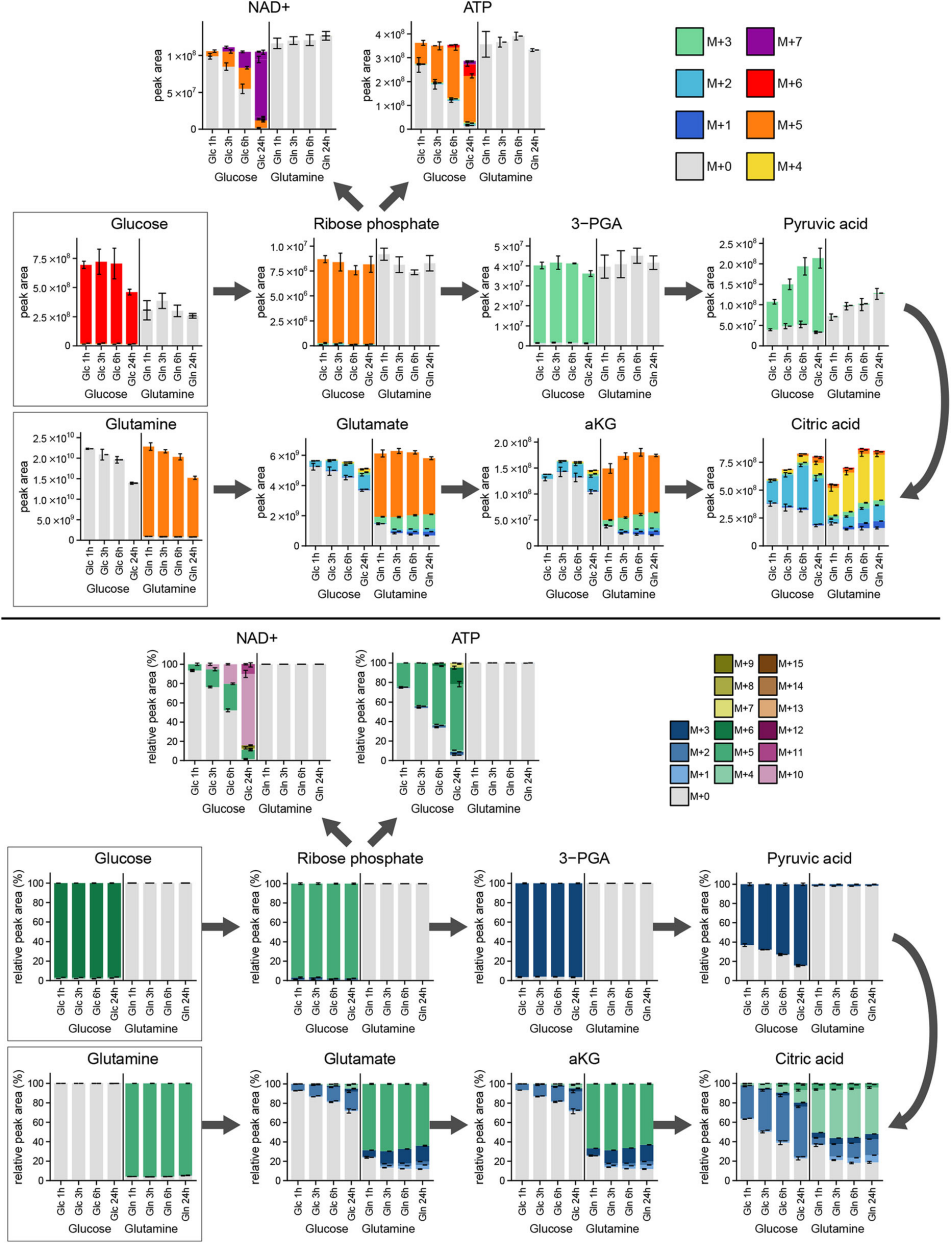
Illustration of tracing experiments processed and visualised with Metabolite AutoPlotter. Plots show the incorporation of ^13^C either from glucose or from glutamine (highlighted on the left side) into some intracellular metabolites of HCT116 cells at different time points ranging from 1 to 24 h. Top: Absolute intensity plots and short colour scale, showing the peak area (arbitrary units). Bottom: relative intensity plots and full colour scale, showing the relative peak area (percentage)

Stable isotope tracing experiments can be processed and visualised as well. Figure 3 shows the results of a ^13^C-tracing experiment performed exclusively to show this feature. For this, HCT116 cells were cultivated in DMEM supplemented with either u-^13^C-glucose or u-^13^C-glutamine for 1 to 24 h and intracellular metabolites were extracted and measured by LC-MS. The isotopologues for tracing experiments should be supplied as “metabolite +1”, ”metabolite +2” and so on. Metabolites up to M+30 can be processed with the application, even though so many masses cannot be shown efficiently. During data processing, the isotopic information is extracted from the metabolite names and all isotopologues are grouped and shown as stacked bars for each condition, indicated by M+0 for the unlabelled species and M+1, M+2, for isotopologes with a mass-shift of one respectively two Dalton. These plots can be shown either in absolute intensities (peak areas, Fig. 3a) or in relative intensities (summed up to 100%, Fig. 3b). Both representations have advantages regarding data interpretation. They can be generated in a single run by repeating the plotting step. Additionally, two different colour scales can be used. The short scale ranging to M+7 is sufficient for showing the central carbon metabolism (Fig. 3a). The longer gradient is based on a stepped rainbow with 5 colour blocks in 3 shades, being able to show up to M+15 allowing an instant overview over the number of isotopologues (Fig. 3b). As an example, the high number of isotopologues in NAD+ cannot be resolved with the short colour scale, while the long scale reveals that it contains mainly M+5 and M+10, from incorporating ribose-phosphate. Figure 3 further shows what can be expected from biology: Some metabolites are labelled exclusively from glucose or glutamine, whereas both tracers contribute to tracing in the TCA-cycle intermediates. With the time course progression, the labelling in most of the metabolites increases as well.

### Natural isotopic abundance correction

The presence of multiple stable isotopes (as ^13^C, ^15^N, ^34^S, ^18^O, ^2^H) in the metabolites can make the interpretation of stable isotope tracing experiments cumbersome. The heavy isotopes introduced by the tracing experiment need to be separated from isotopes being present naturally. The carbon-13 isotope occurs with a frequency of approximately 1.1%. For a 3-carbon molecule like pyruvate, the naturally occurring M+1 isotopologue has a frequency of about 3%, and therefore, the natural abundance of ^13^C makes not much of a difference. However, for a 10-carbon molecule such as adenosine triphosphate (ATP), the M+1 isotopologue will be found with an intensity of 10% relative to M+0 and should not be neglected. It is therefore very important that researchers correct the natural abundance of stable isotopes when performing stable isotope tracing experiments, particularly for large molecules. Often researchers perform experiments with no tracer to show the naturally occurring components. This is however an unnecessary burden given that we can computationally correct for the natural abundance of stable isotopes. We would argue that the computational correction is actually more precise given that the natural abundance of stable isotopes is very consistent across samples.

**Fig. 4:**
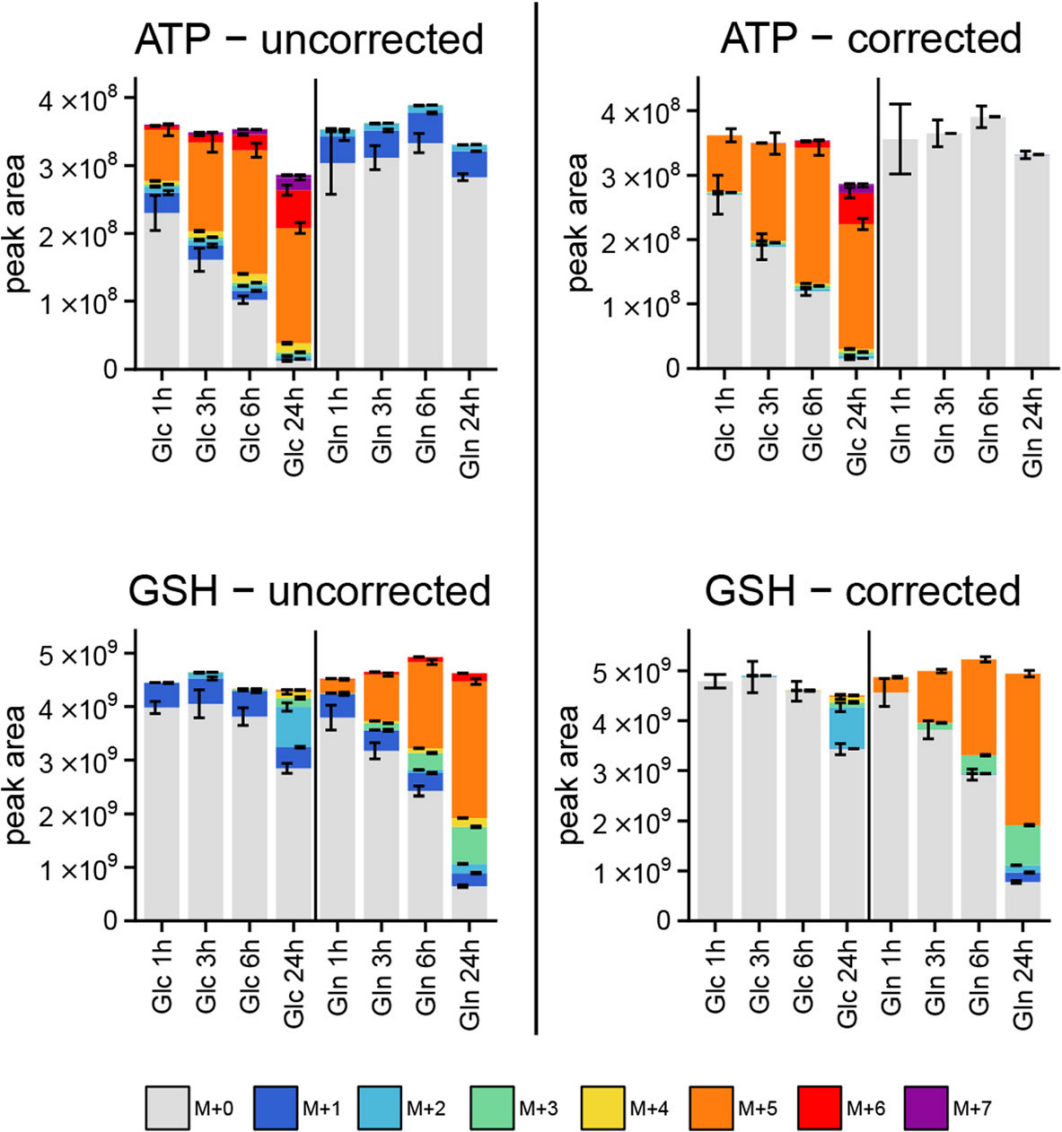
Illustration of the natural abundance correction. The left panels show peak areas (arbitrary units) of ATP and GSH (glutathione), of the same experiment as in Fig. 3, before the natural abundance correction was performed. The interpretation of the results is difficult, particularly when no unlabelled reference is reported. The right panel shows the same compounds after the correction, here the amount of ^13^C incorporation can be deduced much easier. For the legend, see Fig. 3, top

AutoPlotter provides an option for natural abundance correction by integrating the AccuCor package [13]. The user needs to supply the sum-formulae for the unlabelled metabolites, the type of the tracer used (^13^C, ^15^N or ^2^H) and its isotopic purity. If the sum formulae are not already included in the inputs, they can be uploaded later. Figure 4 shows a comparison before and after the correction for ATP (C_10_H_16_N_5_O_13_P_3_) and glutathione (GSH) (C_10_H_17_N_3_O_6_S). In both metabolites, there is a high proportion of M+1 visible (dark blue) and also M+4 (yellow) in ATP. This complicates the interpretation as the reader needs to subtract these contributions in its head, which is impossible without an unlabelled reference sample. After the correction (Fig. 4, right panels) both contributions disappear, revealing an unlabelled state for ATP after ^13^C-glutamine tracing and GSH for the first 6 h of ^13^C-glucose labelling.

### Data safety

The application is hosted at shinyapps.io to benefit from the high-quality standards of the RStudio-team. Users should be aware that the environment is not HIPAA compliant, so confidential data (e.g. non-anonymised patient information) should be removed from the data prior to submission. Beyond that, we make sure that the uploaded data are safe. Intermediary data are stored transiently in a temporary folder on the server, after the session closes data will be deleted. This can also be done by the user pressing a button. We do not have access to the temporary folders; neither do we have the interest or time to check other researchers’ results. Once we published the source code, users can run the tool locally or set up their own servers.

### Limitations and performance

Whilst we worked hard to design the tool to be as open and flexible as possible, we are aware that it cannot be used to address all potential questions. To avoid too high memory usage, the maximum size for the uploaded data is limited to 25 MB, but this should be sufficient for most experiments. There are only 3 essential levels of information needed: the compounds, the samples and their measured intensities. When the compound name is not unique, the retention time (RT) should be supplied additionally to merge these two, and for natural abundance correction, the sum formulae are needed. The complete requirements for the inputs are reported in Table 1.

The generated graphs only allow a discrete *x*-axis, so it is not possible to plot scaled numerical axes as times or concentrations. Also, the names of the conditions shown in the plots need to be different, so this does not allow for grouped plots. This could be realised with more complex or multiple input files for the sample descriptions but would also increase the risk of user errors.

Further, the natural abundance correction as it is implemented currently is limited to elements occurring in nature (C, H, N, O, S), so it cannot be used to correct GC-MS experiments containing Si, Cl or Br.

Beyond that, there are no limitations regarding the number of compounds, replicates or conditions, as long as the memory lasts. We have processed untargeted experiments with over 1000 compounds flawlessly. The performance depends on the number of conditions, the number of replicates and the type of plots being generated. Nevertheless, in most situations, creating the sample-table and adjusting the design is the most time-consuming step and typically takes longer than the time needed to generate the plots. Plotting 250 compounds (5 conditions and 3 replicates) roughly needs 1 min, which nearly doubles when multi-plot overview pages are generated. This is a dramatic performance boost compared to manual plotting in which one could probably generate a maximum of 3 or 4 plots in a minute.

## Discussion

Metabolite AutoPlotter was designed to conveniently process and visualise quantified metabolite data. A wide range of pre-processed input formats (compound-intensity tables) can be used and the design of the plots can be easily adapted to personal preferences. This includes graph types, plotting order, colours, statistics, size, file-format, font sizes, font directions and some more.

Installing and running complex software can be an obstacle for potential users; therefore, we decided to create a web-based application using shiny. With this, the analysis can be performed in a web-browser and results can be downloaded as a zip file. In line with this, more and more shiny applications are published currently, helping with the analysis of different kinds of data [14–20]. Other obstacles can be too strictly defined input formats; therefore, we aimed to keep the inputs as open and well described as possible.

AutoPlotter automatically generates identical plots for all the compounds in the dataset containing data. The plots can be used to be shown in presentations or publications and used to rapidly screen the results and discuss the findings with colleagues, to better understand the data and design follow-up experiments. We are not aware of any other software or webpage that performs similar tasks. It seems as most of the software published do help with the first steps of the data processing as peak integration, compound identification, and data-matrix creation or to identify differences in the (most often untargeted) datasets [1, 3]. VANTED [21, 22] or Cytoscape [23] allow the plotting of multiple compounds, but their primary intention is to put the results into a pathway context, showing multiple compounds at once. Also, they require strictly defined input formats. “PlotsOfData” [17] or “BoxplotteR” [18] are other online tools, enabling the generation of detailed plots (e.g. adding confidence intervals), but here, each and every compound needs to be imported separately. MetaboAnalyst [24, 25], Workflow4metabolomics [26] or XCMS online [27, 28] offer complete solutions starting from the raw data and performing statistical analysis, but even there, it is difficult or not included at all to produce plots for individual compounds.

With its simplicity, this tool aims primarily at researchers new to this field, or running metabolomics experiments as side projects and to targeted analysis where the researcher knows the identity of its compounds. However, it could also be useful for metabolomics core facilities to instantly deliver good quality summaries towards clients while saving manpower and time. For untargeted metabolomics with more than 1000 compounds, it might be more meaningful to identify relevant features first and plot only these compounds, even though it is feasible to plot so many compounds at once.

Although AutoPlotter’s main purpose is the visualisation of metabolite data, it is not limited to this field and could be employed in theory in other fields where similar data (multiple observations over a few conditions) are generated, e.g. proteomics, transcriptomics or drug responses to cell panels. AutoPlotter is under ongoing development and new features will be added in time based on the feedback from the community. Ongoing efforts include the incorporation of clustering to identify compounds with similar responses and normalisation using internal standards.

## Conclusions

Here, we present Metabolite AutoPlotter, a user-friendly application to generate high-quality plots from complex data, in a short time. Automating the data processing and visualisation offers some dramatic improvements over manual processing. It is significantly faster and allows researchers to spend more time interpreting the results or to perform follow-up experiments. Further, this eliminates potential copy-and-paste errors or tedious repetitions when properties need to be changed. It also enables the researcher to consider the full dataset and not just a handful of metabolites that would be plotted manually. Therefore, novel insights might be found in metabolites that would have been overlooked otherwise. Finally, defined tabular outputs generated by automated processing can help to store the data generated in internal databases or to be shared using external repositories with other researchers [29, 30].

## Supplementary information

**Additional file 1.** Detailed description of the exports in the results folder.

**Additional file 2.** Demodata to reproduce the plots shown in the figures.

## Data Availability

The online version can be used freely without registration; the source code to run the application locally may be published in the near future on GitHub. Detailed instructions and demo data used can be downloaded from the start page of the tool. The data needed to reproduce the plots shown in the figures is included in the supplement.

## Abbreviations

ATP: Adenosine triphosphate
DMEM: Dulbecco’s modified Eagle’s medium
GC-MS: Gas-chromatography mass spectrometry
GSH: Glutathione
ICA: Independent component analysis
LC-MS: Liquid chromatography mass spectrometry
m/z: Mass to charge ratio
NAD+: Nicotinamide adenine dinucleotide
PCA: Principal component analysis
PLS-DA: Partial least square discriminatory analysis
R: R-scripting language
RT: Retention time
SD: Standard deviation
TCA-cycle: Tricarboxylic acid cycle = citric acid cycle = Krebs cycle
