# Supplementary material for: Metabolite AutoPlotter - an application to process and visualise metabolite data in the web browser
Source: Cancer Metab. 2020 Jul 10;8:15. doi: 10.1186/s40170-020-00220-x (PMC7350678; doi:10.1186/s40170-020-00220-x)
Supplement: Supplementary file 1 — Additional file 1. Detailed description of the exports in the results folder. [file 40170_2020_220_MOESM1_ESM.pdf]

METABOLITE AUTOPLOTTER - EXPORTED FILES

1. Folder "Inputs"

This folder contains a summary of the imported data. This can be used to reproduce the analysis at a later time point or double check the imported data.

- **1_Input_sorted.csv –** contains a restructured export from the used input, cleaned for samples not used and empty samples. This file can be imported again as "Tracefinder" input.
- **2_imported_Sampletable.xlsx** – contains a copy of the sampletable that was imported for the processing. Together with the above file, or the original dataset the processing can be repeated.
- **3_Metabolite_Summary.xlsx** – lists the features of the detected metabolites, as the number of observations, the retention time, the mass, formula and adducts (when supplied with the input). This can be used to double-check the identifications or create compound libraries matching previous experiments.
- **4_Condition_Summary.xlsx** – contains the summary of the defined conditions, as their order, replicate counts and colours. Colours are also translated to RGB values, for quantitative experiments, to fully reproduce the colours outside, e.g. within Microsoft Office products.

2. Folder "Plots"

This folder contains all the generated plots, a separate legend and the summary pages (when added). Note that special characters (e.g. < > ; ?) are converted automatically to characters being readable by the file-system, so the exact spelling of the files may differ from the uploaded names of the metabolites.

3. Folder QC

This folder contains the result of the quality control.

- **QC-Plot.pdf** – shows the quality control plot. Note that for performance reasons this plot is only generated when it was shown in the "Quality control" feature within Step2.
- **"Relative Area of Replicates.xlsx"** – contains the datapoints shown in the QC-plot (for quantitative experiments) or "**Relative Area Sum of Isotopes.xlsx"** – contains the same data for tracing experiments. Here all the isotopologues are summed up, so the quality control is performed with the total pool size. Both files contain 2 sheets:
  - "Relative_Areas" with the fold changes of between the replicates within the same condition. Here the mean for each metabolite in one condition is 1.
  - "Log2_Areas", contain these values log2 transformed, as shown in the QC-plot, with a mean close to 0.

4. Folder “Tables”

This folder contains the results of the data processing stored in different tables for various purposes.

4A - For quantitative experiments:

- **Export_Prism_Columns.xlsx –** contains the data structured in a way that allows to import these data in column style into GraphPad Prism.
- **Export_Summarised.xlsx –** contains the data in a summarized structured, containing the averaged values (AVG) the standard deviation (SD), the standard error of the mean (SEM) and the sample counts.
- **Table_for_Statistics_[…].xlsx –** contain the processed data in a way that each metabolite is represented by one row, with the different conditions in columns. This allow further calculations on the data, e.g. calculation of statistics. Here “Table_for_Statistics_all_reps” includes all imported conditions. When technical and experimental replicates are defined an additional file “Table_for_Statistics_combined_reps” is generated in which the technical replicates are combined (as shown in the plots). Further this document contain 2 sheets, “original” with the data as it is, and “missings imputed” were missing values are replaced with half of the minimal intensity.

4B - For tracing experiments:

- **“Export_Prism_grouped_abs.xlsx” & “Export_Prism_grouped_rel.xlsx”** – contain the absolute intensities or relative values (as percent) of the isotopologues. These files can be imported and plotted with GraphPad Prism using the grouped template. When natural abundance correction was performed these tables will contain the corrected values.
- **“Export_Summarised_abs.xlsx” & “Export_Summarised_rel.xlsx”** contain the averaged values (AVG) the standard deviation (SD), the standard error of the mean (SEM) and the sample counts, for both the absolute intensities or relative values (as percent). Please note that for the relative data zeros need to be kept within the conditions, so the counts include missing values as well.
- **“Table_for_Statistics_all_reps.xlsx” –** contain the processed data in a way that each metabolite is represented by one row, with the different conditions in columns. This allow further calculations on the data, e.g. calculation of statistics.
- **“Sum_formulas.csv”** - contains the extracted sum formulas used for the natural abundance correction
- **“Natural_abundance_correction.xlsx”** – contains the inputs and the results of the natural abundance correction, when it was performed.
  - “Original” contains the input in a structure needed for the Accucor correction, note that Conditions and replicates are separated by “_._” in order to separate this later.
  - “Corrected” contains the corrected intensities
  - “Normalized” contains the relative intensities
  - “PoolAfterDF” contains the total pool size (with all isotopologues summed up)

See https://rdrr.io/github/XiaoyangSu/AccuCor/man/natural_abundance_correction.html) for details.
